# Prognostic relevance of HER2/neu in acute lymphoblastic leukemia and induction of NK cell reactivity against primary ALL blasts by trastuzumab

**DOI:** 10.18632/oncotarget.7344

**Published:** 2016-02-12

**Authors:** Sebastian P. Haen, Benjamin J. Schmiedel, Kathrin Rothfelder, Bastian J. Schmied, Truong-Minh Dang, Nora Mirza, Robert Möhle, Lothar Kanz, Wichard Vogel, Helmut R. Salih

**Affiliations:** ^1^ University Hospital Tuebingen, Department for Oncology, Hematology, Immunology, Rheumatology and Pulmonology, Tuebingen, Germany; ^2^ Interfacultary Center for Cell Biology, Department for Immunology, Tuebingen, Germany; ^3^ Clinical Collaboration Unit Translational Immunology, German Cancer Consortium (DKTK) and German Cancer Research Center (DKFZ), Tuebingen, Germany

**Keywords:** acute lymphoblastic leukemia, HER2/neu, trastuzumab, rituximab, ADCC

## Abstract

The epidermal growth factor receptor HER2/neu is expressed on various cancers and represents a negative prognostic marker, but is also a target for the therapeutic monoclonal antibody Trastuzumab. In about 30% of cases, HER2/neu is expressed on acute lymphoblastic leukemia (ALL) cells and was proposed to be associated with a deleterious prognosis. Here we evaluated clinical data from 65 ALL patients (HER2/neu^+^, n = 17; HER2/neu^−^, n = 48) with a median follow-up of 19.4 months (range 0.6-176.5 months) and observed no association of HER2/neu expression with response to chemotherapy, disease free or overall survival. *In vitro*, treatment of primary ALL cells (CD20^+^HER2/neu^+^, CD20^+^HER2/neu^−^ and CD20^−^HER2/neu^−^) with Rituximab and Trastuzumab led to activation of NK cells in strict dependence of the expression of the respective antigen. NK reactivity was more pronounced with Rituximab as compared to Trastuzumab, and combined application could lead to additive effects in cases where both antigens were expressed. Besides providing evidence that HER2/neu expression is no risk factor in ALL patients, our data demonstrates that HER2/neu can be a promising target for Trastuzumab therapy in the subset of ALL patients with the potential to improve disease outcome.

## INTRODUCTION

Cytogenetic and surface marker subtyping of leukemia has gained significant importance in diagnosis and treatment of both acute myeloid (AML) and acute lymphoblastic leukemia (ALL). The characterization of genetic mutations and aberrant surface marker expression facilitates the assessment of disease prognosis and serves to guide individual therapy [1, 2]. In the recent years, immunotherapeutic approaches with antibodies have improved the treatment of malignant diseases in general and of ALL in particular. Several strategies using monoclonal or bispecific antibodies have been clinically evaluated leading to a change of treatment paradigm in ALL. The humanized monoclonal antibody Rituximab and the bispecific antibody Blinatumomab, directed to CD20 [3] and CD19 [4], respectively, which stimulate anti-tumor activity of cytotoxic lymphocytes, are meanwhile incorporated into standard ALL therapy. Hence, these and other novel antibodies now represent important strategies to specifically and efficiently target otherwise treatment-refractory diseases [3–7].

Rituximab mediates its effects, among others, by inducing apoptosis, complement dependent cytolysis (CDC) and triggering the Fcγ receptor IIIa (FcγRIIIa, CD16) that mediates antibody dependent cellular cytotoxicity (ADCC) [8]. Increasing evidence indicates that the induction of ADCC in particular of natural killer (NK) cells constitutes a major mechanism that accounts for the clinical effects of Rituximab and Trastuzumab treatment [9, 10]. NK cells are cytotoxic lymphocytes which play an important role in immunosurveillance and represent components of innate immunity. They mediate cellular cytotoxicity and release cytokines (i.e., NK cells constitute the major source of interferon gamma (IFN-γ) in antitumor immunity [11] that shape adaptive immune responses [12]). Owing to their pronounced capacity to mediate ADCC, they largely contribute to the success of antitumor antibodies [13, 14]. In general, NK cells kill target cells with low/absent expression of HLA Class I (“missing-self”) and stress-induced expression of ligands for activating NK receptors (“induced-self”) [15]. A balance of activating and inhibitory signals mediated by multiple different receptors determines whether NK-cell responses are initiated or not [13].

In epithelial tumors, especially in breast and ovarian cancer [16–18], overexpression of the transmembrane receptor tyrosine kinase HER2/neu (c-erb-B2) was originally associated with a deleterious prognosis due to its involvement in oncogenic transformation and metastatic potential [19]. Also in ALL, HER2/neu is expressed in about 30% of cases with B cell precursor origin [6, 20, 21], while blasts of mature B-ALL or T-ALL do not display HER2/neu. Alike in epithelial tumors, an association of HER2/neu expression with chemotherapy resistance and worse clinical outcome was suggested for patients with ALL [21, 22]. Of note, as of now, no long-term follow-up data are available regarding the prognostic relevance of HER2/neu expression in ALL.

For targeting HER2/neu the humanized monoclonal antibody Trastuzumab (Roche, Basel, Switzerland) is legally approved for the treatment of patients with breast cancer and gastric adenocarcinoma that overexpress HER2/neu [23, 24]. This antibody inverted the dismal disease prognosis for epithelial malignancies with HER2/neu expression. Moreover, combination with the second HER2/neu blocking antibody Pertuzumab proved enhanced clinical efficacy leading to the incorporation of this antibody combination into the standard of care for breast carcinoma patients [25].

After binding to its target antigen, Trastuzumab blocks receptor-mediated signalling resulting in reduced tumor growth and angiogenesis and enhances sensitivity to cytotoxic agents when combined with chemotherapy [23, 26]. Moreover, Trastuzumab also induces ADCC, which contributes to its effects upon application to cancer patients [27, 28]. However, the mechanisms by which Trastuzumab mediates its effects in ALL therapy and their potential synergistic effects when combined with other antibodies that are approved for ALL treatment have not been evaluated yet. Notably, Trastuzumab has also shown some clinical efficacy when applied as a monotherapy for treatment of relapsed or refractory HER2/neu^+^ ALL [6].

In this study, we evaluated long-term clinical follow-up data up to 15 years for patients with ALL stratified according to HER2/neu expression in order to determine the applicability of HER2/neu as a prognostic marker in ALL. Furthermore, we evaluated the capacity of Trastuzumab, alone or in combination with Rituximab, to induce NK cell ADCC against ALL blasts.

## RESULTS

### Patient characteristics

We retrospectively analyzed patient records and flow cytometry data of 170 patients diagnosed with ALL at our institution between 1997 and 2011 (Table 1). In 65 cases (mature B-ALL, n = 8 (12%); c-ALL, n = 27 (42%); precursor B-ALL, n = 23 (35%); T-ALL, n = 7 (11%)), data on HER2/neu expression on ALL blasts were available, comprising 17 patients (26%) with HER2/neu surface expression and 48 patients (74%) without. The subtypes of ALL within the HER2/neu^+^ and HER2/neu^−^ patients are provided in Table 1. At initial diagnosis, patients had a median age of 40 years (range 17-83 years). Patients with HER2/neu^+^ ALL were significantly younger than patients with HER2/neu^−^ ALL (median 36 vs. 42 years, p = 0.01). The patient cohort comprised 25 women (38%) and 40 men (62%) with no significant difference between the 2 groups (4 women (24%) and 13 men (76%) vs. 21 women (44%) and 27 men (56%) with HER2/neu^+^ and HER2/neu^−^ ALL, respectively; p = 0.14). Both groups were also balanced with regard to ALL risk stratification according to the criteria of the German ALL study group [29, 30] with the study cohort comprising 8 patients with high risk and 9 patients with standard risk disease in the HER2/neu^+^ group, and 26 patients with high risk and 20 patients with standard risk ALL in the HER2/neu^−^ group (p = 0.8). In two patients, disease risk could not be assessed due to missing data.

**Table 1:** Patient characteristics

Patients	all		HER2/neu^+^		HER2/neu^−^	
Patients total	n = 170					
HER2/neu status	n = 65	100 %	n = 17	26 %	n = 48	74 %
Female	n = 25	38 %	n = 4	24 %	n = 21	44 %
Male	n = 40	62 %	n = 13	76 %	n = 27	56 % p = 0.14
**Age at diagnosis**						
median	40 years		36 years		42 years	
range	17-83 years		20-61 years		17-83 years	p = 0.01*
**Diagnoses**						
mature B-ALL	n = 8	12 %			n = 8	16 %
c-ALL	n = 27	42 %	n = 9	53 %	n = 18	38 %
precursor B-ALL	n = 23	35 %	n = 8	47 %	n = 15	31 %
T-ALL	n = 7	11 %			n = 7	15 %
**Risk Stratification**						
standard	n = 28	43 %	n = 8	47 %	n = 20	42 %
high	n = 35	54 %	n = 9	53 %	n = 26	54 % p = 0.8
not determined	n = 2	3 %			n = 2	4 %

All but one patient received induction chemotherapy treatment (Table 2); the oldest patient (83 years) refused therapy and, hence, was excluded from analyses of sensitivity to chemotherapeutic treatment as well as relapse rates and disease free survival (DFS). Allogeneic hematopoietic cell transplantation (HCT) was performed in 31 patients (48%) with 9/17 patients (53%) in the HER2/neu^+^ and 22/48 patients (46%) in the HER2/neu^−^ group (p = 0.77). 28 patients received one allogeneic graft, in 3 patients a second transplantation was performed after disease relapse (Table 2).

**Table 2:** Therapy and survival

**Initial therapy**			
induction chemotherapy	n = 64	99 %	
no therapy	n = 1	1 %	
**Allogeneic HCT**	n = 31	48 %	
HER2/neu^+^	n = 9	53 %	
HER2/neu^−^	n = 22	46 %	p = 0.77
one HCT	n = 28	43 %	
two HCT	n = 3	3 %	
HER2/neu^+^	n = 1	6 %	
HER2/neu^−^	n = 2	4 %	p = 0.78
**Response to induction chemotherapy**			
blast reduction			
HER2/neu^+^	n = 4	23 %	
HER2/neu^−^	n = 12	25 %	p = 0.90
refractory disease			
HER2/neu^+^	n = 2	12 %	
HER2/neu^−^	n = 5	10 %	p = 0.92
**Refractory to salvage therapy**			
HER2/neu^+^	n = 2 (of 4)	50 %	
HER2/neu^−^	n = 7 (of 12)	58 %	p = 0,88
**Relapse**			
HER2/neu^+^	n = 7	41 %	
HER2/neu^−^	n = 20	42 %	p = 0.95
**Death**			
HER2/neu^+^	n = 11	65 %	
HER2/neu^−^	n = 30	63 %	p = 0.94
**Survival**			
**OS**	**median [months]**	**range [months]**	
HER2/neu^+^	19.7	3.5 − 176.5	p = 0.88
HER2/neu^−^	19.3	0.6 − 171.6	hazard ratio 0.916
**DFS**			
HER2/neu^+^	11.5	0.6 − 176.5	p = 0.99
HER2/neu^−^	10.3	1.0 − 171.6	hazard ratio 1.058

Abbreviations: DFS: disease free survival; HCT: hematopoietic cell transplantation; OS: overall survival

From 13 patients out of the cohort, ALL blasts were available for functional experiments (6 women and 7 men). Five patients had CD20^+^HER2/neu^+^ ALL (4 with c-ALL, 1 with precursor B-ALL), four CD20^+^HER2/neu^−^ ALL, and four CD20^−^HER2/neu^−^ ALL (both 2 with c-ALL and precursor B-ALL each). Median age at initial diagnosis of these patients was 31 years (range 21-64 years) comparable to the patient cohort analyzed for clinical endpoints (p = 0.42).

### Clinical follow-up

**Figure 1:**
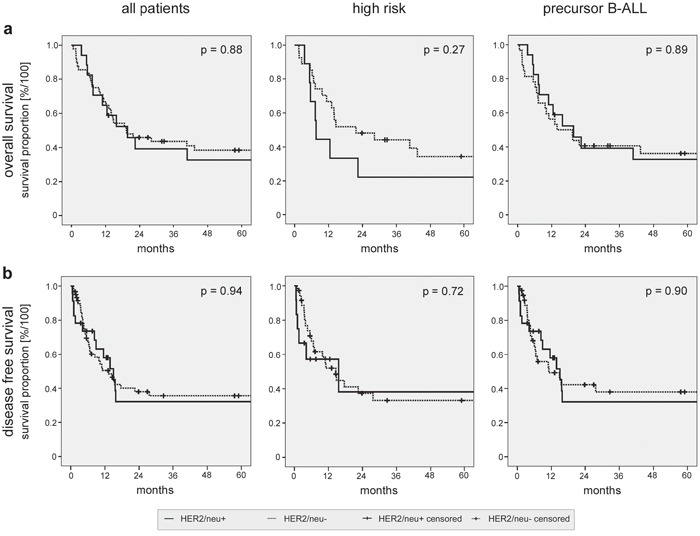
Kaplan-Meier survival analyses of the patient cohort and subgroups Overall **a.** and disease free survival **b.** were analyzed for the complete patient cohort (left panels). Subgroup analyses were performed for patients with high risk disease (middle panels) or with precursor B-ALL only (right panels). Log rank analyses comparing patients with HER2/neu^+^ and HER2/neu^−^ ALL are provided.

Patients were followed for a median of 19.4 months (range 0.6 - 176.5 months) until death or last follow-up examination (Table 2). At the end of follow-up, 24 patients (6 HER2/neu^+^, 18 HER2/neu^−^) were alive and in complete remission (CR), 41 patients (11 HER2/neu^+^, 30 HER2/neu^−^, p = 0.94) had died. One, three and five year overall survival (OS) was 64.6%, 42.5% and 36.9%, respectively. Median OS was 19.7 months and 19.3 months in patients with HER2/neu^+^ and HER2/neu^−^ ALL, respectively, and did not differ significantly (p = 0.88, hazard ratio 0.916) (Fig. 1). Also, no statistically significant difference was observed for DFS (11.5 vs. 10.3 months, p = 0.99, hazard ratio 1.058). Relapse of disease occurred overall in 27 patients of our cohort with 7 patients belonging to the HER2/neu^+^ and 20 to the HER2/neu^−^ group with no statistically significant association with HER2/neu expression (p = 0.95). Primary refractory disease (albeit blast reduction possible) was observed in 4 and 12 patients in the HER2/neu^+^ and the HER2/neu^−^ group, respectively (p = 0.90). Resistance to initial chemotherapeutic treatment was observed in 2/17 patients with HER2/neu^+^ ALL and 5/47 patients with HER2/neu^−^ ALL (p = 0.92). In first relapse, 2/4 patients with HER2/neu^+^ ALL and 7/12 patients with HER2/neu^−^ ALL did not respond to salvage chemotherapy (p = 0.88).

**Figure 2:**
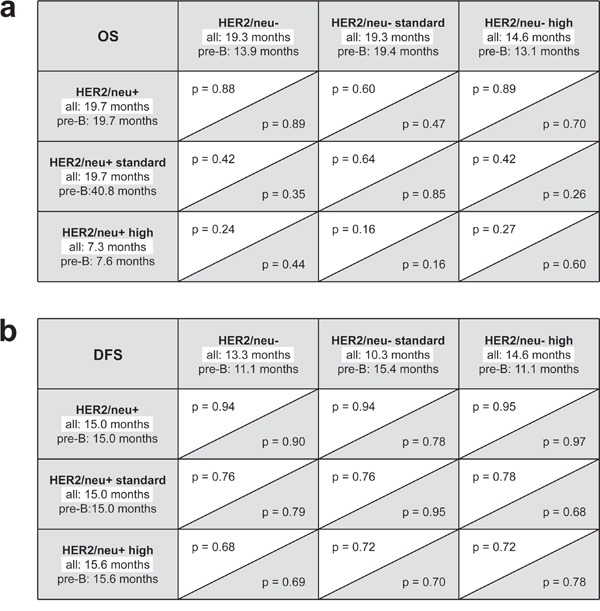
Log rank analyses in patient subgroups **a.** Subgroup analysis of overall survival (OS). **b.** Subgroup analyses of disease free survival (DFS). Statistical levels of significance are provided for the complete patient cohort (white background) and the subgroup of patients with precursor B-ALL only (gray background).

Next, subgroup analyses (Figs. 1 and 2) with the following cohorts were performed: (I) High risk disease in HER2/neu^+^ vs. HER2/neu^−^ ALL. (II) Standard risk disease in HER2/neu^+^ vs. HER2/neu^−^ ALL. Again, no significant differences in OS and DFS were observed. The detailed results of the survival analyses in the different patient subgroups are provided in Fig. 2.

Since patients with HER2/neu^+^ ALL were significantly younger than patients with HER2/neu^−^ ALL, we also adjusted analyses of OS and DFS for the age groups below and above 40, 50 and 60 years. However, again, no significant differences of OS and DFS in the two groups were found (data not shown).

Finally, we analyzed our patient cohort after excluding patients with mature B- and T-ALL, since no HER2/neu expression was detected on mature B-ALL and T-ALL blasts in our and antecedent analyses of other investigators [20–22]. This was to exclude a potential bias towards the group of patients with HER2/neu^−^ ALL. Again, no significant differences in DFS (15.0 vs. 11.1 months, respectively, p = 0.90) and OS between patients with HER2/neu^+^ and HER2/neu^−^ disease (19.7 vs. 13.9 months, respectively, p = 0.89) were observed (Figs. 1 and 2). For patients with precursor B-ALL, results of the same subgroup analyses as performed for the full patient cohort are provided with Fig. 2. In summary, no significant association of HER2/neu expression on ALL blasts and sensitivity to induction therapy, course of the disease and disease progression was observed in our patient cohort.

### Stimulation of NK cell reactivity by Trastuzumab and Rituximab

Rituximab is meanwhile routinely employed in the treatment of ALL patients with CD20-expressing blasts and mediates its clinical efficacy largely by induction of ADCC [31]. With Trastuzumab, another approved antibody is available which can target ALL blasts including CD20 negative cases for ADCC [27, 32]. Notably, Trastuzumab was already employed in a clinical ALL trial [6]. Based on the mechanisms involved in antibody therapy of epithelial or lymphoid cancers [33, 34], we first set out to comparatively analyze the capacity of Trastuzumab and Rituximab to induce anti-leukemia reactivity of NK cells. Thereby we aimed to exclude that the Fc parts of Trastuzumab and Rituximab *per se* differ with regard to their capacity to trigger CD16 in the absence of target cells, which in turn would result in a differential effect on the reactivity of NK cells independently of binding to the target antigens. As clustering is necessary to induce profound CD16 signaling, an assay system was implemented in which the antibodies were immobilized on plastic to facilitate receptor triggering [35]. The absence of target cells in this assay system also allowed to exclude potential effects of other immunoregulatory molecules expressed by target/effector cells which may interfere with the analysis of effects of CD16 stimulation. To this end, polyclonal NK cells of single healthy donors (pNKC) were cultured on immobilized Rituximab, Trastuzumab and a combination of both, and NK activation was determined after 24 h.

**Figure 3:**
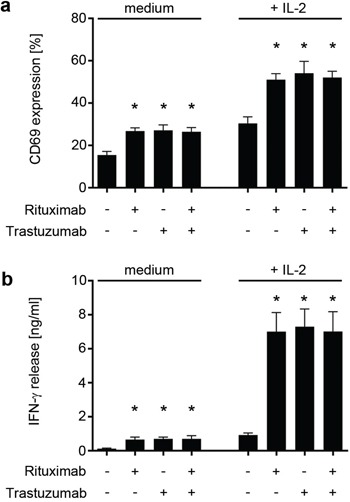
Trastuzumab and Rituximab comparably induce NK cell activation Polyclonal NK cells of healthy donors were cultured for 24 hours without or with 25 U/mL IL-2 (+ IL-2) in the absence (medium) or presence of Trastuzumab, Rituximab or a combination of both (10 μg/ml each) after immobilization to plastic. Combined data of 8 independent experiments in the absence and presence of IL-2 are shown. **a.** The percentage of CD69-positive CD56^+^CD3^−^ NK cells as determined by FACS is indicated. **b.** The production of IFN-γ was determined by ELISA. Statistically significant results are indicated by *, the respective p values are provided in the results section.

Analysis of CD69 levels as marker for NK activation revealed that expression was significantly upregulated upon incubation on Rituximab (p < 0.0001), Trastuzumab (p < 0.0001) and their combination (p < 0.0001). No statistically significant differences were observed between the two antibodies or their combination compared to the effect of single antibodies. Additional presence of interleukin (IL)-2, which served to mimic a generally augmented state of NK reactivity, further enhanced the effects of CD16 stimulation on NK activation (p = 0.0007, p = 0.0006, p < 0.0001 for Rituximab, Trastuzumab or their combination, respectively), but again without significant differences between Rituximab, Trastuzumab and their combination (Fig. 3a). In line, IFN-γ release was clearly induced upon incubation on Rituximab, Trastuzumab and their combination without detectable differences between the two antibodies or the combination compared to the effect of single antibodies, and this held true in the absence (p = 0.005, 0.02 and 0.002, respectively) and presence (p = 0.0008, 0.001 and 0.0005, respectively) of IL-2 (Fig. 3b).

### Induction of ADCC and cytokine release of NK cells in response to ALL blasts upon Trastuzumab and Rituximab treatment

**Figure 4:**
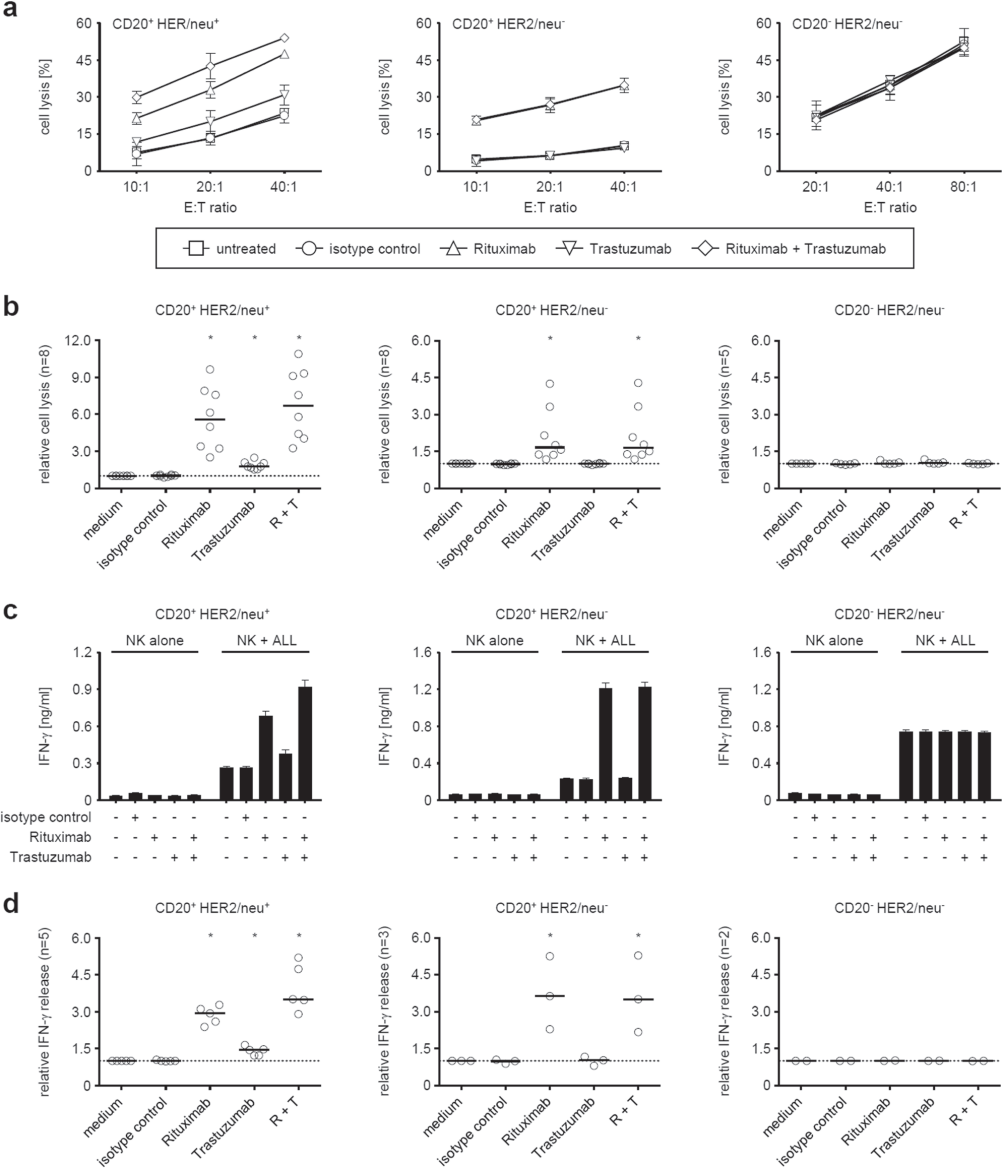
Induction of NK reactivity against primary ALL cells by Trastuzumab and Rituximab Leukemic cells of patients with CD20^+^HER2/neu^+^ (left panels), CD20^+^HER2/neu^−^ (middle panels), and CD20^−^HER2/neu^−^ ALL blasts (right panels) were cultured with allogeneic NK cells in the absence or presence of Rituximab and Trastuzumab (alone or in combination) or an irrelevant isotype control (all 10 μg/ml) as indicated. **a, b.** Target cell lysis was determined by 2 hour BATDA europium assays. **c, d.** IFN-γ levels in culture supernatants were analyzed after 24 hours by ELISA. (a, c) Representative results of single experiments (symbols with error bars representing means and SD of triplicate measurements each), (b, d) results obtained with ALL blasts of the indicated number of patients and median (−) of results obtained at an effector to target cells ratio of 20:1 in cytotoxicity assays (b) and 1:1 in analyses of cytokine release (d)). To enable overall statistical analysis, individual data sets were normalized by defining target cell lysis or cytokine release by NK cells in response to ALL blasts in the absence of antibodies as 1. Statistically significant results are indicated by *, the respective p values are provided in the results section.

Next, we aimed to determine the capacity of Trastuzumab to stimulate NK cell reactivity against ALL cells and compared its effects to that of Rituximab. To this end, we employed primary CD20^+^HER2/neu^+^, CD20^+^HER2/neu^−^ and CD20^−^HER2/neu^−^ ALL blasts (non-cultured PBMC from ALL patients with a percentage of leukemic cells > 80%) in cytotoxicity assays with pNKC. Natural cytotoxicity of pNKC against target cells was dependent on the employed effector:target cell ratio and varied highly among different experiments, which can be attributed to the differing mismatches between patients and allogeneic healthy NK donors that translate in differences between activating or inhibitory signals and thus lytic activity in the absence of the therapeutic antibodies. As expected, neither antibody affected lysis of CD20^−^HER2/neu^−^ ALL cells. When CD20^+^HER2/neu^−^ target cells were employed, only Rituximab induced significant (p < 0.001) ADCC. With CD20^+^HER2/neu^+^ target cells, both Rituximab and Trastuzumab significantly (both p < 0.001) increased lysis by allogeneic NK cells (Fig. 4a and 4b). Notably, despite the fact that Trastuzumab and Rituximab comparably stimulated NK cells via CD16 in the absence of target cells (Fig. 3), a generally weaker effect of Trastuzumab compared to Rituximab was observed. Treatment with both antibodies led to significantly (compared to incubation with Rituximab alone, p < 0.05) increased target cell lysis despite the fact that either antibody was used in saturating doses in our experiments, indicating that Trastuzumab may cause additive effects when applied together with Rituximab *in vivo* (Fig. 4a and 4b). Similar results were obtained with regard to antibody-induced IFN-γ production. We found that mere presence of the leukemic cells already induced cytokine release by pNKC, and this was, in strict dependence on target antigen expression, significantly enhanced by Trastuzumab and Rituximab (both p < 0.01). The effect of Rituximab was again significantly (p < 0.01) more pronounced than that of Trastuzumab, and a significant (p < 0.01) additive effect was observed with ALL cells expressing CD20 and HER2/neu upon application of both antibodies (Fig. 4c and 4d). Of note, the employed allogeneic NK cells differed in the analyses of IFN-γ induction and cell lysis.

Pertuzumab, another therapeutic HER2/neu^+^ antibody which targets a different epitope than Trastuzumab and is also approved for treatment of HER2/neu^+^ breast cancer [25] has been reported to exert additive effects with regard to ADCC when combined with Trastuzumab [36]. In our experiments, Pertuzumab was found to display a similar capacity to trigger CD16 on NK cells and induce IFN-γ release when compared to Trastuzumab. In functional analyses with primary ALL blasts, Pertuzumab also stimulated NK reactivity albeit to a lesser extent than Trastuzumab. Similar to the combination of Trastuzumab and Rituximab, additive effects were observed upon combined application with Trastuzumab in analyses with allogeneic NK cells only, but not with autologous NK cells, which could be attributable to the low number of experiments in the autologous system (Supplementary Fig. 1). As Pertuzumab has never been evaluated for the treatment of ALL, our study focused on Trastuzumab.

**Figure 5:**
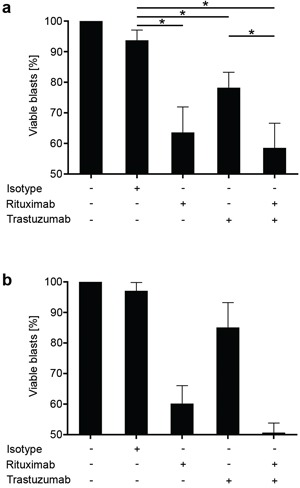
Trastuzumab induces lysis of primary ALL by autologous NK cells PBMC of ALL patients (CD20^+^HER2/neu^+^) were left untreated or incubated with or without 10 μg/ml of Rituximab, Trastuzumab, a combination of both or isotype control (Cetuximab) for 24h in the presence of 10% autologous serum. Then ALL cell lysis by autologous NK cells was determined by FACS. For combined analysis, results of single experiments were calculated as percent of viable cells compared to untreated controls (100%). Combined results obtained in 7 independent experiments **a.** and results of one representative exemplary experiment **b.** are shown. Error bars represent means and SEM (pooled data) or SD (individual data). Statistically significant results are indicated by *, the respective p values are provided in the results section.

In an effort to mirror treatment of leukemia in patients as closely as possible, we then directly employed PBMC of ALL patients *ex vivo* to study the effects of the antibodies on NK cell lysis in an autologous system (Fig. 5). In line with the results obtained in the allogeneic setting, presence of Trastuzumab (p = 0.02) and Rituximab (p = 0.0009) resulted in a significantly enhanced lysis of the ALL cells with a more pronounced effect observed with the CD20 antibody. Of note, combined treatment with both antibodies did, in contrast to the results in the allogeneic setting, not cause a significantly more pronounced lysis as compared to Rituximab treatment alone (p > 0.05).

### Characterization of target antigen expression on ALL cells

**Figure 6:**
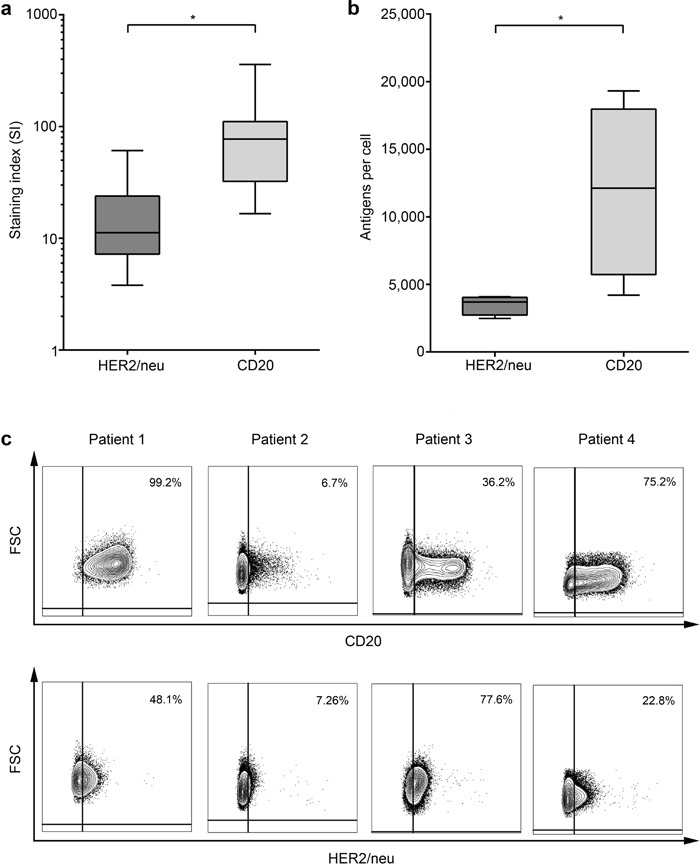
Comparative analysis of CD20 and HER2/neu expression levels on primary ALL blasts HER2/neu and CD20 expression on primary ALL blasts at time of initial diagnosis was determined by flow cytometry. **a.** Staining indices (SI) were calculated using the formula (median fluorescence [positive population] − median fluorescence [negative population] / 2× standard deviation [negative population]). **b.** Absolute quantification of molecule numbers was performed as described in the methods section. Error bars represent means and SEM. **c.** The percentage of CD20 and HER2/neu-positive ALL cells within leukemic cells of individual patients as determined by flow cytometry is shown.

Next we determined whether the observed lower NK-stimulatory capacity of Trastuzumab as compared to Rituximab correlated with expression of the respective target antigens on the leukemic cells. Flow cytometric analysis revealed significantly lower expression levels (Fig. 6a) of HER2/neu as compared to CD20 (staining index (SI) median 11.2 (range 3.1-61.0) and median 70.4 (range 4.5-358.3), respectively; p < 0.01). Notably, HER2/neu and CD20 expression did not differ between patients with c-ALL or precursor-B-ALL in our cohort (SI median 9.0 vs. 15.5, p = 0.53 and 59.4 vs 87.6, p = 0.91, respectively; data not shown).

Next we analyzed the absolute molecule numbers of the target antigens on leukemic cells of 4 patients with CD20^+^/HER2/neu^+^ ALL. While the numbers varied substantially among the individual patients, we observed clearly higher values for CD20 as compared to HER2/neu, which even reached statistical significance despite the low number of samples available for this analysis (mean 11,945 vs. 3,489 molecules per cell, p = 0.005, Fig. 6b). As downregulation of antigens constitutes a potential mechanism by which malignant cells may evade targeted treatment, we also determined the percentage of antigen-positive blasts within single patients. We again observed a substantial inter-individual variation with regard to both antigens, however in this case without a clearly more pronounced positive population for CD20, at least in the small number of cases available for this analysis (Fig. 6c). Due to limited availability of patient material, analysis of ALL subgroups with regard to CD20 and HER2/neu surface molecule numbers was not feasible.

### Influence of Rituximab and Trastuzumab on CDC and monocyte/macrophage reactivity against ALL cells

**Figure 7:**
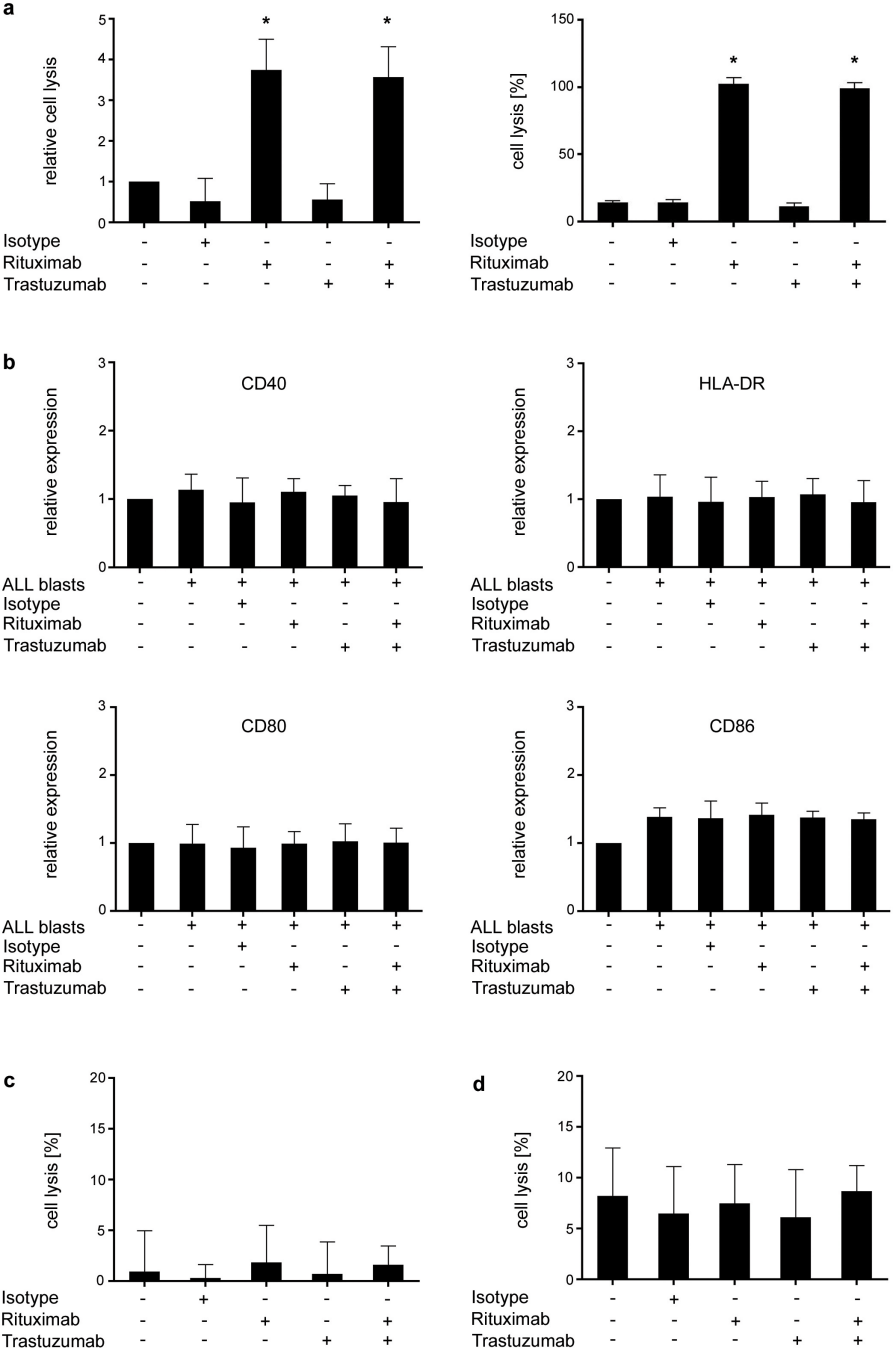
Analysis of the effects of Rituximab and Trastuzumab on CDC and monocyte/macrophage reactivity PBMC of ALL patients (CD20^+^HER2/neu^+^) were left untreated or incubated with or without 10 μg/ml of Rituximab, Trastuzumab, a combination of both or isotype control in the presence or absence of serum **a.** monocytes **b, c.** or macrophages **d**. (a) After 2 hour incubation, CDC was determined by BATDA Europium assays. Results of single experiments were normalized to the medium control. Relative blast lysis was calculated as multiples of medium control. Results obtained in 4 independent experiments (left panel) and representative results of one exemplary experiment (right panel) are shown. Statistics were calculated in comparison with the medium control. (b) Monocyte activation as reflected by upregulation of CD40, CD80, CD86 and HLA-DR was measured by flow cytometry after 16 hours of incubation with or without antibodies or isotype control. Pooled data of 3 independent experiments are shown. Surface antigen expression (MFI) was normalized to untreated monocytes (set to 1) and calculated as changes from baseline expression. Statistics were calculated in comparison with untreated monocytes. (c) Monocyte-mediated lysis of ALL blasts was determined after 2 hour incubation with or without antibodies or isotype control (10 μg/ml each) by BATDA Europium assays. Pooled data of 3 independent experiments are shown. (d) Macrophage-mediated lysis of ALL blasts was determined after 2 hour incubation with or without antibodies or isotype control (10 μg/ml each) by BATDA Europium assays. Pooled data of 3 independent experiments are shown. Error bars represent means and SD. Statistically significant results are indicated by *.

Next, we aimed to evaluate a potential contribution of the complement system in ALL blast lysis mediated by Trastuzumab and Rituximab. In line with previous observations, Rituximab clearly mediated CDC, while, alike reported with epithelial tumors, no CDC against ALL blasts was induced by Trastuzumab (Fig. 7a) [8, 37]. This could also serve to explain the discrepancy between the results observed in the lysis assays conducted in the allogeneic and autologous system, as the latter is performed in the presence of substantial amounts of serum.

Next we studied how treatment with the two antibodies affects reactivity of monocytes and macrophages, which may contribute to clearance of antibody-laden cells [38], against ALL blasts. However, in our *in vitro* analyses we did not observe relevant effects of either antibody: flow cytometric analysis of monocytes in cultures with ALL cells did not reveal alterations of surface molecules associated with activation upon antibody treatment (Fig. 7b). Moreover, neither constitutive lysis of ALL cells by monocytes nor effects of either antibody or their combination on the latter was observed (Fig. 7c). When macrophages were employed as effector cells, some limited constitutive lysis of ALL cells was detected, but again not affected by treatment with either antibody or their combination (Fig. 7d). Notably, antibody-mediated phagocytosis of ALL blasts, which has been reported to contribute to the effects of antibody treatment in ALL [7] was not analyzed in our study.

## DISCUSSION

Prior to the introduction of Trastuzumab into standard of care of HER2/neu^+^ breast cancer and gastric adenocarcinoma, expression of this receptor tyrosine kinase was associated with a deleterious prognosis [23, 24]. Notably, also ALL cells can express HER2/neu, and several studies suggested that expression also represented a negative prognostic marker in this hematopoietic malignancy [21, 22]. However, all previous studies in ALL have been underpowered to determine a valid prognostic significance, and so far no long-term follow-up data were available regarding the prognostic relevance of HER2/neu expression in ALL. We here evaluated data from a heterogeneous patient cohort treated at our institution with a follow-up up to 15 years and did not observe any differences regarding the incidence of primary chemotherapy resistance, relapse rates, DFS or OS. While it needs to be considered that our observations derive from a retrospective analysis of data obtained at a single center, these results nevertheless indicate that in contrast to epithelial tumors, HER2/neu expression does not constitute a prognostic marker for disease course in ALL. Due to the relatively small patient number, we were, however, not able to identify potential subgroups that might experience a different course of disease dependent on HER2/neu expression. Notably, HER2/neu in ALL, alike in epithelial tumors, represents a therapeutic target for treatment with Trastuzumab.

In earlier years, outcome in adult ALL has been improved mainly by optimization of chemotherapy protocols and allogeneic transplantation [39]. More recently, several antibodies have been introduced in the treatment of ALL patients. The T cell engaging bispecific single chain antibody (BiTE) Blinatumomab directed against CD19 was mainly evaluated upon disease relapse or for elimination of minimal residual disease (MRD) [5]. Use of the monoclonal antibody Rituximab has yielded promising clinical results leading to its embodiment in the treatment of de novo ALL [3]. Trastuzumab has also already been evaluated in ALL patients experiencing disease relapse or refractory disease and was applied as monotherapy in a phase-II study [6]. Treatment resulted in a limited overall response rate of 13% in these pretreated patients. To our knowledge, Trastuzumab is currently not utilized in clinical studies for the treatment of ALL (clinicaltrials.gov, accessed on December 15th 2015) and has never been evaluated as component of induction and/or consolidation chemotherapy, despite the fact that better clinical responses may be achieved when combined with conventional chemotherapy due to its dual mechanism of action observed in epithelial cancers: Trastuzumab can both increase chemosensitivity of tumor cells and induce reactivity of immune effector cells [6, 10, 23, 26]. Thus, evaluation of this antibody in the treatment of *de novo* ALL, alone or in combination with Rituximab, appears reasonable. This is supported by our *in vitro* data that upon treatment of ALL blasts Trastuzumab potently induces antitumor activity of NK cells, which play an important role in immunosurveillance and, due to their pronounced capacity to mediate ADCC, largely contribute to the success of antitumor antibodies [13, 14, 40]. In contrast to Rituximab, Trastuzumab did not influence CDC against ALL blasts, and neither Rituximab nor Trastuzumab induced monocyte activation or lysis by monocytes/macrophages in our experimental system. As primary ALL cells rapidly undergo cell death upon *in vitro* culture, potential effects of Trastuzumab, alone or in combination with chemotherapy, on ALL blast apoptosis were not analyzed in our study for technical reasons.

Targeting two different antigens like CD20 and HER2/neu would increase the total amount of antibody bound to target cells, potentially resulting in triggering of more Fc-receptors and/or activation of a greater amount of NK cells as suggested by our data. Notably, induction of NK cell ADCC was less pronounced with Trastuzumab as compared to Rituximab, which can be explained by the lower expression levels/molecule counts of HER2/neu compared to CD20 on the cell surface of ALL blasts observed by us and others [41] and is in line with the fact that induction of ADCC by a given antibody correlates with the expression level of its target antigen [42, 43]. Of note, additive effects upon combined application were observed in the allogeneic system only, but not in our analyses in the autologous setting. Besides a potential selection bias caused by the low number of available CD20^+HER2/neu+^ samples, the CDC mediated by Rituximab may have influenced the same, as CDC may have contributed to lysis in the autologous system, in which, for technical reasons in contrast to the allogeneic setting, substantial amounts of serum were present.

Combined application of more than one therapeutic antibody is currently evaluated in multiple clinical trials/disease entities in order to utilize different biological mechanisms and, hence, overcome tumor escape through antigen loss. For example, Rituximab treatment is combined with inhibition of vascular endothelial growth factor (Bevacizumab) or antibody-drug conjugates targeting CD30 (Brentuximab vedotin). Both Rituximab and Trastuzumab are also evaluated in combination with immune checkpoint inhibiting antibodies (clinicaltrials.gov, accessed on December 15^th^ 2015). Our data indicate that enhancing ADCC induction by application of two antibodies targeting different antigens on the same target cell may also warrant clinical evaluation.

The same held true for induction of IFN-γ release, the latter constituting an important mechanism by which NK cells shape adaptive immune responses [12]. Moreover, interferons plays a pivotal role in the elimination phase of the cancer immunoediting process [44, 45]. Among others, IFN-γ is crucial for enhancing tumor cell elimination by macrophages and increases MHC-I and MHC class II expression on APC, maturation of dendritic cells and antigen presentation [46, 47]. Since our experiments revealed that combination of Trastuzumab and Rituximab may result in additive effects, Trastuzumab treatment represents a feasible strategy not only in patients with CD20^−^HER2/neu^+^ ALL which cannot be treated with Rituximab, but also for combined application with Rituximab in CD20^+^HER2/neu^+^ ALL. Moreover, determination of expression levels and percentages of target antigen-positive cells may provide a rationale for individually adapted treatment with the two antibodies. Notably, we did not evaluate the potential influence of the CD16 V158F polymorphism reported to affect ADCC in our experimental system. However, as NK cells from different donors were used in each particular experiment, the influence of this polymorphism on the combined results of the multiple analyses conducted in our study would be expected to be limited.

With regard to a further evaluation of the validity of the immunostimulatory effects of Trastuzumab in ALL in future studies it needs to be considered that utilization of mouse models may be compromised due to the fact that (antibody-induced) killing mechanisms of NK cells differ in mice and humans [48, 49]. Moreover, significant differences between human and murine Fc and FcγRIIIA structure exist, and mouse FcγR are distributed and bind human IgG1 differently than their human counterparts [48, 50]. It was recently also shown that in xenograft mouse models routinely used to characterize therapeutic monoclonal antibodies, neutrophils are sufficient to mediate IgG-induced antitumor activities [51], which is in stark contrast to observations that in humans NK cells, among the various immune effector cells that are activated upon antibody treatment, are the main mediators of ADCC [14]. Thus, the murine immune system does not truly reflect the situation in humans. Models employing cancer cells and human cells transferred to immunodeficient mice are, among others, hampered by contamination from cellular populations other than NK cells, which often affects tumor growth, and the shorter half-life time of human NK cells in mice, where they are lost rapidly [52]. For these reasons, evaluation of the immunostimulatory effects of Trastuzumab in mouse models to substantiate and improve the information conferred by our data may turn out to be difficult. Notably, the results obtained by *ex vivo* analyses in the autologous system directly using PBMC of leukemia patients that contain leukemic cells and effector cells in a “realworld human situation” are, in our opinion, most suitable to provide valid information. Moreover, as Trastuzumab has already been successfully used in clinical ALL trials [6], application to HER2/neu^+^ ALL patients and/or combined application with Rituximab in CD20^+^HER2/neu^+^ double positive patients, in our view, could also directly be evaluated in humans.

In summary, the data presented in this study provide evidence that HER2/neu expression does not impact chemotherapy sensitivity, DFS or OS of ALL patients. However, HER2/neu may constitute a promising therapeutic target for Trastuzumab, and application, alone or in combination with other antibodies, in patients with HER2/neu^+^ ALL may represent a promising strategy to take advantage of both the immunomodulatory and chemotherapy-modifying effects of Trastuzumab that warrants further clinical validation.

## MATERIALS AND METHODS

### Acquisition of samples from patients and healthy donors

Peripheral blood mononuclear cells (PBMC) of ALL patients (obtained at the time of diagnosis before therapy) and healthy donors were isolated by density gradient centrifugation (PAA Laboratories, Pasching, Austria). Informed consent was obtained from all patients and healthy donors in accordance with the Declaration of Helsinki, and the study was performed according to the guidelines of and approved by the institutional ethics committee.

### Flow cytometric analysis of HER2/neu and CD20 expression on primary ALL blasts

HER2/neu and CD20 expression on ALL blasts was determined by flow cytometry with PE-conjugated antibodies (BD Biosciences, Franklin Lakes, New Jersey, USA) and counterstaining with CD19-FITC conjugates (BD Biosciences). HER2/neu-PE, CD19-PE and CD20-PE were diluted 1:2 and hence used in clinically validated saturating concentrations. Detection was performed using a FACSCalibur flow cytometer (BD Biosciences). Staining indices were calculated using the formula (median fluorescence [positive population] − median fluorescence [negative population] / 2× standard deviation [negative population]).

### Quantification of surface expression of HER2/neu and CD20

Quantitative determination of cell surface antigens was performed using the QIFIKIT (Dako, Hamburg, Germany) according to the manufacturer's protocol as previously described [53]. In brief, ALL cells were incubated with CD20-PE and HER2/neu-PE antibodies (BD Biosciences) validated for diagnostic purposes followed by a goat-anti-mouse-PECy7 conjugate (Biolegend, San Diego, California, USA, 1:100). Leukemic cells were selected by counterstaining for CD19 or CD19/CD34 with CD19-FITC and CD34-APC conjugates (BD Biosciences). Antigen density on positive cells was corrected for unspecific binding using a PE-labelled isotype control (BD Biosciences).

### Preparation of pNKC

The pNKC were generated as described previously [54]. Briefly, non-plastic adherent peripheral blood mononuclear cells (PBMC) from healthy individuals were regularly obtained twice weekly and incubated with irradiated RPMI8866 feeder cells (ratio 4:1) in the presence of IL-2 (25 U/ml) for 10 days. Subsequently, purity of NK cells was determined by flow cytometry. Functional experiments were performed with NK cells (CD56^+^CD3^−^) of more than 90% purity. Flow cytometry antibodies were CD56-FITC and CD3-PeCy5 (BD Biosciences, both diluted 1:25).

### NK cell activation

As marker for NK cell activation (1 × 10^6^ pNKC per condition), upregulation of CD69 was analyzed by flow cytometry. pNKC from at least 8 different single donors were used and not pooled in individual experiments. Specific fluorescein conjugates (CD69-PE, BD Biosciences, diluted 1:25) were used for FACS analysis. Where indicated, IL-2 was added to further increase NK cell activity at 25 U/ml.

### Cytotoxicity assays

Cytotoxicity of NK cells was analyzed by BATDA Europium release assays as described previously [54]. In brief, PBMC of patients (> 80% leukemia cell count) were labeled with BATDA (Wallac Oy, Turku, Finland), washed, and placed in 96-well round-bottomed plates at 5,000 per well followed by addition of therapeutic antibodies (Rituximab, Trastuzumab and Pertuzumab) or isotype control (Cetuximab) where indicated and addition of pNKC. After incubation for 2 h, 20 μl of supernatant per well were removed and mixed with 200 μl DELFIA Europium Solution (Wallac Oy). Cytotoxicity was quantified by measuring the fluorescence of the Europium TDA chelates using a time-resolved fluorometer (VICTOR, Wallac Oy), and results are shown as means of triplicate measurements. Maximum release was determined from target cells lysed in 1% Triton X-100. Percentage of lysis was calculated as follows: 100 × (experimental release − spontaneous release) / (maximum release − spontaneous release).

### Determination of cytokine levels

Cytokine production (IFN-γ) was measured using the specific ELISA antibody set from Thermo Scientific (Waltham, Massachusetts, USA) according to the manufacturer's instructions. Cytokine concentrations are presented as means of triplicate measurements. Where indicated, IL-2 was added at 25 U/ml.

### Analysis of ALL blast lysis by autologous NK cells

The influence of Rituximab, Trastuzumab and Pertuzumab (therapeutic antibodies, all from Roche, Basel, Switzerland) on lysis of ALL blasts by autologous NK cells was determined by FACS as described previously [54]. In brief, PBMC of ALL patients were incubated in the presence of 10% autologous serum with or without therapeutic antibodies or isotype control (Cetuximab) as indicated (all at 10 μg/ml). The employed concentration of 10 μg/ml was selected to utilize saturating concentrations and based on the consideration that this dose mirrors the real-life situation in patients upon clinical application of recommended dosing. After 24 h, ALL cells were selected by staining for CD19^+^ or CD19^+^CD34^+^ (CD19-FITC, BD Biosciences; CD34-APC, BD Biosciences), and dying or dead cells were identified by 7-Actinoaminomycin (7-AAD, BD Biosciences) positivity. CD19-FITC was diluted 1:200, CD34-APC 1:25, 7-AAD was diluted 1:200. Analysis of equal assay-volumes was ascertained by adding unlabeled standard calibration beads to every sample, which allowed for the determination of the number of ALL blasts that had vanished from the culture. Percentage of specific lysis was calculated as follows: 100-[(7-AAD negative cells in respective antibody treatment/7-AAD negative cells in control) × 100]. The results are shown as means of triplicate measurements.

### Analysis of CDC

Complement-mediated lysis of leukemic cells upon antibody treatment was assessed by 2-hour BATDA Europium assays. In brief, leukemic cells were labeled with BATDA as described above and incubated in the presence or absence of antibodies or isotype control (all at 10 μg/ml in serum-free medium) for 1h at 37°C. After adding serum from healthy donors (final dilution of 1:3), 5,000 cells per well were incubated for 2 h. Then lysis was determined by measuring fluorescence of Europium TDA chelates as described above.

### Analysis of monocyte activation by ALL blasts and antibodies

Monocytes were isolated from PBMC of healthy donors using the Monocyte Isolation Kit II (Miltenyi Biotec, Bergisch-Gladbach, Germany) according to the manufacturer's protocol.

Monocytes were incubated overnight (16 hours) under pyrogen-free conditions with ALL blasts (1:1) and antibodies (10 μg/ml each). Fluorochrome staining against CD14 (Alexa Fluor 700; 1:200), CD40 (Pe/Cy7; 1:20), CD80 (FITC; 1:20), CD86 (Brilliant violet 605; 1:20), and HLA-DR (Brilliant violet 711; 1: 40; all obtained from Biolegend), as well as Aqua LiveDead (Thermo Fisher, Waltham, Massachusetts, USA). Detection was performed using a Fortessa flow cytometer (BD Biosciences).

### Analysis of ALL blasts lysis by monocytes and macrophages

Monocytes were isolated from buffy coats of healthy donors as described above. For macrophage differentiation, the cells were resuspended in cell growth medium (RPMI 1640 medium (Sigma-Aldrich, Munich, Germany) supplemented with 10% heat-inactivated FCS (GE Healthcare, Solingen, Germany), 5 mM L-glutamine (Life Technologies, Carlsbad, California, USA) and 1 % Pen Strep (Thermo Fisher)) and differentiated to macrophages by incubation with 25 ng of GM-CSF (PeproTech, Hamburg, Germany) per milliliter for 7 days. Non-adherent cells were thoroughly removed by washing at day 5.

After confirming differentiation to macrophages by microscopy, cells were employed in cytotoxicity assays on day 7. To this end, ALL cells (5,000/well) were incubated in the presence or absence of antibodies or isotype control (all at 10 μg/ml) for 1h, then added to macrophages (ratio 1:25). After 2h, lysis of ALL cells was determined by measuring fluorescence of Europium TDA chelates using the BATDA Europium assay described above.

To determine monocyte cytotoxicity against ALL cells, BATDA labeled ALL cells were incubated in the presence or absence of antibodies or isotype control (all at 10 μg/ml) for 1h. Then monocytes were added (ratios 10:1 to 80:1, for ratios 1:10 to 1:40 data not shown) and lysis of ALL cells was determined after 2h using the BATDA Europium assay described above.

### Statistics

Statistical tests were performed using SPSS Version 22 Software (2013, IBM Corporation, Ehningen, Deutschland), as well as GraphPad Prism Version 6 (2012, GraphPad Software Inc., La Jolla, California, USA). Tests included Student's T tests for analysis of experimental results and Chi-Square-tests as well as Kaplan Meier regression with Mantle Cox log rank analysis for analyses of clinical data. *p*-values < 0.05 were considered statistically significant and are marked in the graphs with an asterisk.

Overall survival (OS) was calculated for all patients from initial diagnosis to death or last follow-up visit. Patients alive at last follow-up visit were censored.

Disease free survival (DFS) was calculated from initial diagnosis or from diagnosis of relapse to repeated relapse or last follow-up visit. Patients without disease relapse at death or last follow-up visit were censored.

## SUPPLEMENTAL FIGURE


