# Supplementary material for: Prognostic relevance of HER2/neu in acute lymphoblastic leukemia and induction of NK cell reactivity against primary ALL blasts by trastuzumab
Source: Oncotarget. 2016 Feb 12;7(11):13013–30. doi: 10.18632/oncotarget.7344 (PMC4914338; doi:10.18632/oncotarget.7344)
Supplement: Supplementary file 1 [file oncotarget-07-13013-s001.pdf]

SUPPLEMENTARY FIGURE

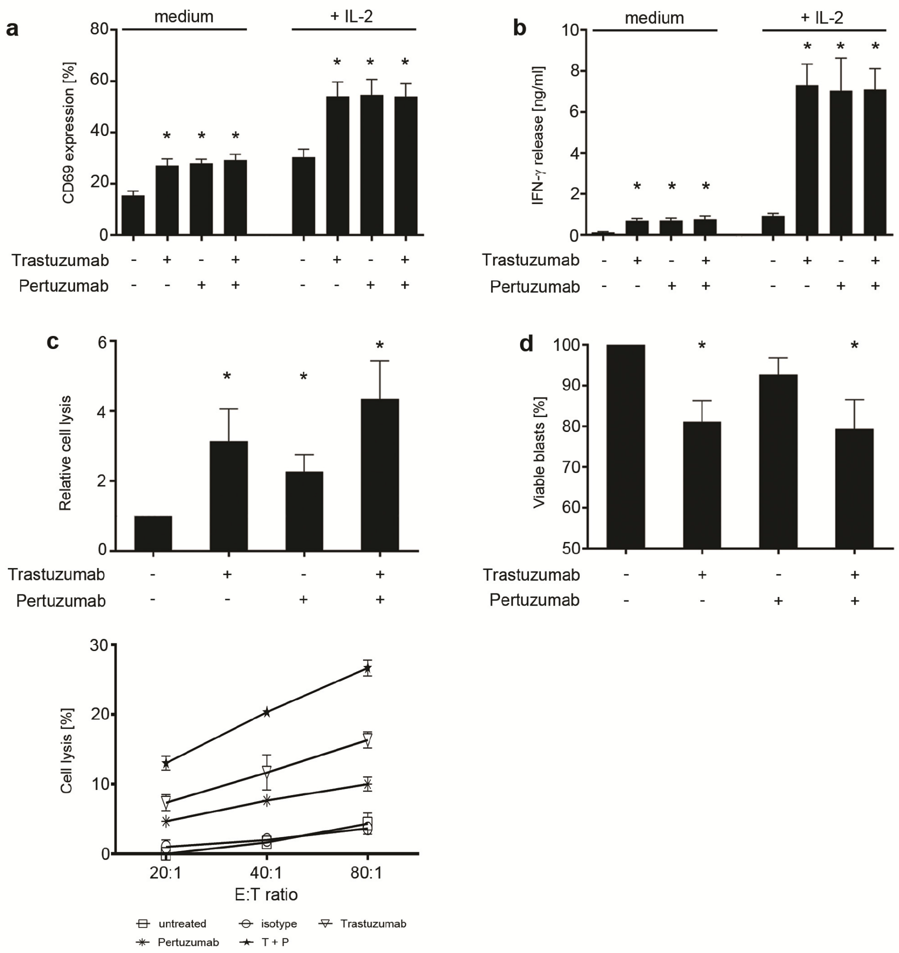

Supplementary Figure S1: Effects of Pertuzumab on NK cell activation and target cell lysis of primary ALL blasts. a,b. pNKC were cultured for 24 hours without (medium) or with 25 U/mL IL-2 (+ IL-2) in the absence or presence of Trastuzumab, Pertuzumab (10 µg/ml each) or a combination of both (T + P) after immobilization to plastic. Combined data of 8 independent experiments in the absence and presence of IL-2 are shown. **a.** The percentage of CD69-positive NK cells was determined by FACS. Expression was significantly upregulated following incubation on Trastuzumab (w/o IL-2: $p < 0.0001$; + IL-2: $p = 0.0007$), Pertuzumab (w/o IL-2: $p < 0.0001$; + IL-2: $p = 0.0009$) and the combination of both antibodies (w/o IL-2: $p < 0.0001$; + IL-2: $p = 0.0004$) as compared to medium. **b.** Determination of IFN-γ release by ELISA also revealed significant effects upon incubation with Trastuzumab (w/o IL-2: $p = 0.02$; + IL-2: $p = 0.001$), Pertuzumab (w/o IL-2: $p = 0.02$; + IL-2: $p = 0.0002$) and the combination of both antibodies (w/o IL-2: $p = 0.008$; + IL-2: $p = 0.001$) as compared to medium. Neither with regard to CD69 expression nor cytokine release, relevant differences were observed between the two antibodies or their combination as compared to the effect of single antibodies. **c.** Primary $CD20^{+}HER2/neu^{+}$ ALL cells were employed in cytotoxicity assays with pNKC in the absence or presence of Trastuzumab, Pertuzumab (alone or in combination) or an irrelevant isotype control (all 10 µg/ml). Combined results (upper panel) obtained in 7 independent experiments at an effector to target cells ratio of 80:1 and exemplary results of one representative experiment (lower panel) are shown. To enable statistical analysis, individual data sets were normalized by defining target cell lysis or cytokine release by NK cells in response to ALL blasts in the absence of antibodies as 1. A significant increase of ALL cell lysis as compared to cells cultured without antibody or in the presence of an irrelevant isotype control was observed with Trastuzumab ($p = 0.01$), Pertuzumab ($p = 0.005$) and the combination of both ($p = 0.01$). Combination of both antibodies showed more pronounced effects as compared to Trastuzumab ($p = 0.01$) or Pertuzumab ($p = 0.005$) monotherapy [36]. **d.** PBMC of ALL patients ($CD20^{+}HER2/neu^{+}$) were left untreated or incubated with or without 10 µg/ml of Trastuzumab, Pertuzumab, a combination of both or isotype control (Cetuximab) for 24h. Then ALL cell lysis by autologous NK cells was determined by FACS. Results of single experiments were calculated as percent of viable cells compared to untreated controls (100%). Combined results obtained in 7 independent experiments are shown. A significant increase of ALL cell lysis as compared to cells cultured without antibody was observed with Trastuzumab ($p = 0.002$) and the combination of both antibodies ($p = 0.01$), but not with Pertuzumab alone ($p = 0.07$). Notably, no relevant additive effects of the combination of both antibodies compared to Trastuzumab alone were observed. Error bars represent means and SEM (pooled data) or SD (individual data). Statistically significant results are indicated by *.
